# Characterization and Cytocompatibility of Collagen–Gelatin–Elastin (CollaGee) Acellular Skin Substitute towards Human Dermal Fibroblasts: In Vitro Assessment

**DOI:** 10.3390/biomedicines10061327

**Published:** 2022-06-04

**Authors:** Nusaibah Sallehuddin, Nur Izzah Md Fadilah, Ng Min Hwei, Adzim Poh Yuen Wen, Salma Mohamad Yusop, Nor Fadilah Rajab, Yosuke Hiraoka, Yasuhiko Tabata, Mh Busra Fauzi

**Affiliations:** 1 Centre for Tissue Engineering and Regenerative Medicine, Faculty of Medicine, Universiti Kebangsaan Malaysia, Cheras, Kuala Lumpur 56000, Malaysia; nusaibahsallehuddin@gmail.com (N.S.); nurizzahfadilah@gmail.com (N.I.M.F.); angela@ppukm.ukm.edu.my (N.M.H.); 2 Department of Surgery, Hospital Canselor Tuanku Muhriz, Universiti Kebangsaan Malaysia, Cheras, Kuala Lumpur 56000, Malaysia; adzimpoh@gmail.com; 3 Department of Food Sciences, Faculty of Science and Technology, Universiti Kebangsaan Malaysia, Bangi, Selangor 43000, Malaysia; salma_my@ukm.edu.my; 4 Biomedical Science Program, Center for Healthy Aging and Wellness, Faculty of Health Sciences, Universiti Kebangsaan Malaysia, Jalan Raja Muda Abd Aziz, Kuala Lumpur 50300, Malaysia; nfadilah@ukm.edu.my; 5 R&D Centre, Biomaterial Group, Nitta Gelatin Inc., 2-22, Futama Yao City, Osaka 581-0024, Japan; yo-hiraoka@nitta-gelatin.co.jp; 6 Laboratory of Biomaterials, Department of Regeneration Science and Engineering, Institute for Life and Medical Science (LiMe), Kyoto University, 53 Kawara-cho Shogoin, Sakyo-ku, Kyoto 606-8500, Japan; yasuhiko@infront.kyoto-u.ac.jp

**Keywords:** CollaGee, genipin, biomaterial, collagen, gelatin, elastin, wound healing

## Abstract

Full-thickness skin wounds have become a serious burden to patients, medical care, and the socio-economic environment. The development of a safe and effective acellular skin substitute that can rapidly restore intact physiological skin is required. Natural bioactive materials including collagen, gelatin, and elastin possess significant advantages over synthetic biomaterials regarding biodegradability and biocompatibility. However, low mechanical strength, a faster biodegradation rate, and thermally unstable biomaterials lead to slow-healing and a high rate of post-implantation failure. To overcome these concerns, naturally occurring genipin (GNP) flavonoids were added to improve the mechanical strength, degradation rate, and thermal properties. Therefore, this study aimed to fabricate and characterize collagen–gelatin–elastin (CollaGee) biomaterials cross-linked with GNP as an acellular skin substitute potentially used in full-thickness wound healing. CollaGee at different ratios was divided into non-cross-linked and cross-linked with 0.1% GNP (*w*/*v*). The physicochemical, mechanical, and biocompatibility properties of CollaGee were further investigated. The results demonstrated that GNP-cross-linked CollaGee has better physicochemical (>50% porosity, pore size range of 100–200 µm, swelling ratio of >1000%) and mechanical properties (resilience and cross-linking degree of >60%, modulus of >1.0 GPa) compared to non-cross-linked CollaGee groups. Furthermore, both cross-linked and non-cross-linked CollaGee demonstrated pivotal cellular compatibility with no toxicity and sustained cell viability until day 7 towards human dermal fibroblasts. These findings suggest that GNP-cross-linked CollaGee could be a promising ready-to-use product for the rapid treatment of full-thickness skin loss.

## 1. Introduction

Wound healing is a highly complex and dynamic process for restoring impaired or injured tissue to its original physiological form. Full-thickness wounds can be divided into acute or chronic based on the wound-healing progress. These wounds are mainly caused by trauma, infections, immobility, or chronic illness [1,2]. Immediate wound management is a realistic approach to improving the rate of healing and minimizing the risk of complications. Dressings and antibiotics are the gold standards for wound-management approaches at the moment. However, ideal wound care has yet to be achieved. In addition, healed wounds develop hypertrophic scars that lead to movement restriction and cosmetic concerns [3,4]. It has been reported that treatment costs are in the range of USD 28.1 to 96.8 billion for 8.2 million Medicare beneficiaries [5]. Hence, a new spectrum in wound-healing technology is essentially necessary. Cutaneous scarring affects 80 million people worldwide, while hypertrophic scarring occurs explicitly in 40–70% of third-degree burn patients [3]. The current wound-care technologies aim to develop an ideal wound dressing to cater to these problems; it must retain moisture, be biocompatible and biodegradable, promote angiogenesis, and completely re-epithelialize [3].

Additionally, among the disadvantages of a collagen–gelatin wound dressing is the formation of scarring-contracted tissue [6,7]. This scenario could be potentially overcome by adding elastin to the biomaterial fabrication as a distinguished strategy. Elastin is not produced immediately after birth; however, its production can be induced by injuries or diseases but is inadequately expressed during the wound-healing process [8]. As a result, the intact elastic fiber network appearance is long delayed after injury, hence reducing the mechanical strength and scar elasticity compared to normal physiological skin [8].

Tissue-engineering technology relies on developing 3D porous biomaterials that provide a microenvironment that resembles the native tissue anatomical structure and proper biodegradation that existed prior to the newly formed tissue. Additionally, it supports the tissue, promotes cell attachment and cell proliferation, and provides signaling molecules to promote tissue regeneration [9]. Moreover, it functions as a carrier for active or therapeutic compounds to the wound sites [8]. A feasible strategy for materials’ hybridization could improve the properties and successfully act as a controlled-release device for the incorporation of biomolecules to expedite wound healing. A 2019 review reported that natural-based biomaterials such as collagen, gelatin, and elastin have been widely used in tissue engineering due to their excellent physicochemical and mechanical properties. In addition, they could also reduce prolonged inflammation and immune rejection post-implantation at the wound site [9].

Existing proteases and enzymes tend to remodel the natural biomaterials in the body. Although natural biomaterials have different stiffness properties, they have relatively low mechanical propensity [10]. Nevertheless, they can be further modified to develop functionalized biomaterials that require only temporary remodeling at the implanted site [11]. However, a single type of biomaterial may not meet all the requirements needed for an optimum wound-healing biomaterial due to its advantages and disadvantages. Therefore, researchers will utilize two or more biomaterials to improve the physicochemical and mechanical properties to obtain biomaterial designs that are intended for a final wound-healing application [12].

The elastin-based biomaterial has gained interest in tissue engineering due to its good biocompatibility, biomechanical, and biodegradation properties in addition to decreasing wound contraction by regulating fibroblast differentiation, elevating dermal regeneration, and enhancing skin elasticity [13,14]. Elastin is the second most common structural component in the extracellular matrix (ECM). It plays an essential role in wound healing and contributes to many living tissues’ elasticity and resilience [15,16]. Elastin peptides modulate the cellular response to induce monocyte chemotactic and fibroblast migration and proliferation, protease production, keratinocyte migration, smooth muscle proliferation, and promotion of an angiogenic phenotype in endothelial cells [17,18,19,20,21,22,23]. The remodeling outcome is dependent upon the favorable response of the host immune system. Native ECM is composed of collagen and elastin; therefore, skin wound-dressing biomaterial should include collagen and elastin as the primary biomaterials [15].

Collagen is the most abundant protein in the ECM, with approximate 30% of total body proteins. Therefore, collagen is a favorable biomaterial for skin wound-healing applications. Collagen contributes to cell attachment, migration, proliferation, and differentiation and gene expression through specific cell receptors and cell-binding sites, and retention, local storage, and delivery of growth factors and cytokines. These attributes play a key role in organogenesis and tissue regeneration [12,24]. Gelatin is also known as a molecular derivative of type I collagen due to several of its advantages compared to its precursor. Firstly, it is a promising material in tissue engineering due to its chemical similarities with ECM in the native tissues. It has functional groups that allow facile chemical modifications with other biomaterials of biomolecules [25]. Gelatin contributes to a balanced hydrophobicity/hydrophilicity of the fabricated biomaterials, resulting in an appropriate release of the bioactive compounds [1]. Its susceptibility to enzymatic degradation can be used to generate controlled release materials.

Furthermore, it is necessary to confer structural stability to the implanted biomaterials by introducing exogenous cross-linking into the molecular structure [26]. Exogenous cross-linking agents stabilize the collagen molecule by forming covalent and hydrogen bonds between the fibers [24]. In addition, the cross-link formation can protect or amend major antigenic receptors and decrease their capacity to bind with antibodies [22]. However, the synthetic cross-linker is commonly portrayed as a potentially toxic substance, leading to switching to naturally derived cross-linkers [27]. Therefore, cross-linking agents that are not detrimental to the biomechanics and biocompatibility of the ECM, such as genipin (GNP), may present a good option [28]. GNP is an aglycone, which is extracted from an iridoid glycoside, called geniposide, from the fruits of *Gardenia jasminoides Ellis*. It is a cross-linking agent with high efficacy, good biocompatibility, and insignificant toxicity, and it inhibits fibroblast-induced contraction [28,29]. The GNP reacts with primary amines through a nucleophilic attack while, simultaneously, the ring opening of polymerization of GNP creates long-range intermolecular cross-linking [29,30]. This long-range intermolecular cross-linking leads to improved mechanical properties and resistance against enzymatic hydrolysis [31]. Therefore, using functional hybrid biomaterials with natural cross-linkers, incorporated for the rapid treatment of full-thickness wound-care management, is worth explaining.

This study aimed to develop a 3D hybrid biomaterial from a combination of collagen (extracted from ovine tendon), Halal gelatin (Nitta Gelatin Inc., Osaka, Japan), and elastin (produced from poultry skin) cross-linked with a natural GNP cross-linker. The biomaterial was fabricated via a freeze-dryer approach followed by physicochemical, mechanical, and cell–biomaterial interaction evaluations.

## 2. Materials and Methods

This study protocol was approved by the Universiti Kebangsaan Malaysia (UKM) Research Ethics Committee (Code No. JEP-2019-424).

### 2.1. Collagen Type I Extraction and Purification

The extraction of collagen type I was performed as previously described by Fauzi et al. with some modifications [32]. Briefly, the freeze-dried ovine tendon was immersed in 0.35 M sterilized acetic acid (Merck, Darmstadt, Germany) for 72 h to dissolve the collagen protein and remove any impurities. The swollen tendons were blended with similar sterilized acetic acid to produce a homogeneous solution at a chilling temperature of 4–8 °C. Then, the blended solution was centrifuged at 5488× *g* at 4 °C for 10 min. The supernatant was collected and precipitated, using sodium chloride (Sigma-Aldrich, St. Louis, MO, USA) overnight prior to dialyzing for 72 h. Next, the collagen was pre-frozen at −80 °C for 6 h and lyophilized to obtain the dried collagen flakes for a final working solution preparation (re-dissolved in 0.35 M sterile acetic acid).

### 2.2. Fabrication of Collagen–Gelatin–Elastin Biomaterials

The mixture of 1.425% (*w*/*v*) collagen stock and 0.7% (*w*/*v*) gelatin solution was stirred gently at two different ratios, of 4 mL:1 mL and 4.5 mL:0.5 mL, for 5 min. Then, 5 mg and 10 mg of elastin powder were added to the 4:1 and 4.5:0.5 ratios, respectively, and gently mixed for 10 min using a stirrer. The mixed solutions were poured into the desired mold and frozen at −80 °C for 6 h followed by freeze-drying for 24–48 h at −56 °C and 5 mTorr of atmospheric pressure. Then, the freeze-dried biomaterials were cross-linked with 0.1% (*w*/*v*) genipin (Fujifilm Wako Pure Chemical Corporation, Shibukawa, Japan) (GNP). Briefly, the GNP solution (0.1% *w*/*v*) was made by mixing crystallized GNP powder in 70% ethanol (EtOH; MERCK, Darmstadt, Germany) at room temperature (22–24 °C). The CollaGee biomaterials were then immersed in 0.1% (*w*/*v*) GNP at room temperature for 6 h followed by washing, using Dulbecco’s Phosphate Buffered Saline (DPBS) (Sigma-Aldrich, St. Louis, MO, USA) three times to obtain 415_GNP and 4510_GNP formulations of GNP-cross-linked CollaGee biomaterials. Non-cross-linked biomaterials for each corresponding ratio were used as control, namely, 415_NC and 4510_NC. The cross-linked CollaGee biomaterials were then freeze-dried for 24–48 h for further analysis.

### 2.3. Gross Appearance and Resilience

The gross appearance of the fabricated CollaGee biomaterials was captured using a digital camera (Nikon, Tokyo, Japan). The resilience was determined by placing 300 g of weight on the wet biomaterials for 10 min. Then, the biomaterials were immersed in distilled water for 2 min. The thickness areas of the pre-compression and post-rehydration were documented. The images were analyzed using Image J software (Version 1.53; National Institutes of Health, Bethesda, MD, USA). Resilience experiments were performed in triplicate. The resilience was experimentally determined using the following formula:

Resilience = (Ab/Ar) × 100

where Ab is the thickness area before compression and Ar is the thickness area after rehydration.

### 2.4. In Vitro Biodegradation

The biodegradability of the CollaGee biomaterials was evaluated by incubating each sample in 0.0006% (*w*/*v*) collagenase type I (Worthington, Columbus, OH, USA) prepared in phosphate buffered saline (PBS) (Sigma-Aldrich, St. Louis, MO, USA). The initial weight of the biomaterials (Wi) was recorded accordingly, and the biomaterials were placed in the incubator at a temperature of 37 °C. Every 24 h, the biomaterials were washed with PBS and freeze-dried for 12–24 h. Experiments were performed in triplicate. The remaining biomaterials were weighed (Wf) and the weight loss (%) was calculated by using the following formula:

Biodegradation rate = (Wi − Wf)/t

where Wi is the initial weight, Wf is the final weight, and t is the time.

### 2.5. Swelling Testing

The swelling ratio analysis was performed by rehydrating the biomaterials at 37 °C in DPBS for 1 h. Before rehydration, the biomaterials were weighed in a dry form. Experiments were performed in triplicate. The swelling ratio was calculated as follows:

Swelling ratio = (Ws − Wd)/Wd × 100

where Ws is the swollen weight and Wd is the dry weight.

### 2.6. Cross-Linking Degree Analysis

A ninhydrin assay was used to determine the degree of cross-linking. A 10 mg sample for each group was soaked in 200 µL of a 10× ninhydrin 2% (Sigma-Aldrich, St. Louis, MO, USA) working solution and boiled for 2 min at 100 °C. After cooling down, 200 µL of 95% ethanol was added to the solution. The absorbance was read at 570 nm. Experiments were performed in triplicate. The cross-linking degree was calculated as follows:

Cross-linking degree = (Anc − Ac)/Anc × 100

where Anc is the absorbance of the non-cross-linked and Ac is the absorbance of the cross-linked.

### 2.7. 3D Microstructural Analysis

The morphological evaluation was performed on the surface and cross section of the CollaGee biomaterials. A field emission scanning electron microscope (FESEM), operated at 15 kV, was used to observe the cross-section microstructure of the CollaGee biomaterials. The pore size distribution was measured using Image J software (Version 1.53; National Institutes of Health, Bethesda, MD, USA). The porosity was evaluated by measuring the weight difference between pre- and post-submersion in 99.5% ethanol for 18 h. Porosity experiments were performed in triplicate. The porosity was calculated using the following equation:

Porosity = (Ws − Wd)/(ρH20 × V) × 100

where Ws is the submerged weight, Wd is the dry weight, ρ is the density of 99.5% ethanol, and V is the volume of the biomaterial.

### 2.8. Chemical Characterization

The chemical characterization of the CollaGee biomaterials was performed using Fourier-transform infrared spectroscopy (FTIR). The CollaGee biomaterials (1 mm^3^) were analyzed, and the spectral data were recorded by a PE spectrum 100 FTIR spectrometer (PE, Waltham, MA, USA) at a wavelength range of 700 to 4000 cm^−1^. The absorbance peaks were analyzed to identify the chemical structure and chemical changes due to cross-linking process. The chemical composition of the CollaGee biomaterials was analyzed using energy-dispersive X-ray (EDX) to identify elements within the fabricated CollaGee and to provide quantitative information. The crystallographic structure analysis of the fabricated CollaGee biomaterials was performed by X-ray diffraction (XRD).

### 2.9. Thermal Stability

The denaturation temperatures of the CollaGee biomaterials were determined by differential scanning calorimetry (DSC) under a nitrogen atmosphere at a heating rate of 10 °C/minute up to 120 °C. In contrast, thermogravimetric analysis (TGA) of the CollaGee biomaterials was performed at a heating rate of 10 °C/minute under a nitrogen atmosphere of 35 °C to 800 °C.

### 2.10. Mechanical Testing

The mechanical strength of the CollaGee biomaterials was measured in a dry condition at room temperature. The instrument was fitted with 50 N of load transducer at a crosshead velocity of 0.05 mm/minute. Experiments were performed in triplicate.

### 2.11. Isolation and Culture of Human Dermal Fibroblasts

HDFs were obtained as redundant tissue from six consenting patients and processed as previously described by Fauzi et al. [33]. Briefly, 3 cm^2^ of skin samples were cleaned, minced, and digested via a dual-enzymatic process using 0.6% collagenase (Worthington, Columbus, OH, USA) type I at 37 °C for 4–6 h followed by treatment with trypsin-EDTA (Sigma-Aldrich, St. Louis, MO, USA) for 10 min. The cell suspension was then centrifuged and resuspended with a co-culture medium containing an equivalent amount of Epilife (Gibco, Waltham, MA, USA) supplemented with 5% fetal bovine serum (FBS) (Sigma-Aldrich, St. Louis, MO, USA) and F12: DMEM (Gibco, Waltham, MA, USA) containing 10% FBS. The skin cell suspensions containing human epidermal keratinocytes (HEK) and HDFs were seeded in a polystyrene culture plate and placed at 37 °C in an incubator supplied with 5% CO_2_. The medium was changed three times per week. HDFs were dissociated by differential trypsinization after the cell had achieved 70–80% confluency. HDFs were cultured in a 75 cm^2^ T-flask with F12: DMEM containing 10% FBS.

### 2.12. Cell Toxicity Assessment of CollaGee Biomaterials

The LIVE/DEAD^®^ Cell Viability Assay (Invitrogen, Waltham, MA, USA) was used to analyze the cytotoxic effect of the CollaGee biomaterials according to the manufacturer’s protocol. Briefly, HDFs were cultured on the CollaGee biomaterials for 24 h, incubated with 2 mM calcein AM and 4 mM EthD-1 in DPBS for 30 min, and washed with DPBS. Subsequently, observation of the cultures was performed using a Nikon A1R-A1 confocal laser scanning microscopy. The live cells were stained with green colors while the dead cells were stained with a red color. The morphological features of the cells on the CollaGee biomaterials were observed using a scanning electron microscope (SEM).

### 2.13. Cell Viability and Attachment Evaluation

The most reliable method in determining the cell viability of HDFs is by using 3-(4,5-dimethylthiazol-2-yl)-2,5-diphenyltetrazolium bromide (MTT) (Invitrogen, Waltham, MA, USA) staining based on the color changes of tetrazolium yellow powder into insoluble, dark-purple crystals of formazan. This occurrence happens only in living cells where succinate dehydrogenase enzymes are present in their mitochondria [34]. Formazan crystal was then disintegrated in organic solvents such as DMSO and its absorbance was recorded by a spectrophotometer. The resulting absorbance value corresponded to the formazan concentration, which corresponded to the number of living cells.

This test was performed in a direct method. For this purpose, the CollaGee bioscaffolds were firstly sterilized using sterile 70% ethanol. A total of 2 × 10^4^ of HDFs was seeded on the top of the CollaGee bioscaffolds in 24-well plates and incubated for 1, 3, and 7 days. The cell-seeded bioscaffolds were cultured and incubated at 37 °C under a 5% CO_2_ atmosphere to allow the attachment of the cells to the bioscaffolds. Then, 200 µL of F12: DMEM (Gibco, USA) containing 10% FBS (Sigma-Aldrich, St. Louis, MO, USA) was added to each well and treated for 1, 3, and 7 days. Next, 300 µL of F12: DMEM and 30 µL of MTT reagent (5 mg/mL in PBS) (Sigma-Aldrich, St. Louis, MO, USA) were added to each sample followed by incubation at 37 °C for 4 h for MTT formazan formation. Negative controls were made by adding 300 µL of F12: DMEM to 30 µL of MTT solution without the presence of cells. Then, 225 µL of the medium was discarded from each well by using a micropipette. A total of 100 µL of DMSO (Sigma-Aldrich, St. Louis, MO, USA) was added to each well to dissolve the formazan crystals. The absorbance was measured at 540 nm using a microplate reader (Power Wave XS; Bio-TEK Instruments, Winooski, VT, USA). The percentage of viability was determined using the following equation:

$$\mathrm{Cell}\;\mathrm{viability}\;(\%)=\frac{\mathrm{At}}{\mathrm{Au}}\times 100$$

where At is absorbance treated and Au is absorbance untreated.

The cell attachment assay of HDFs was determined by Trypan blue dye exclusion assay. Briefly, HDFs were cultured directly on the CollaGee bioscaffolds and incubated for 24 h. The cultured media were centrifuged for 5 min at 500 rpm (Hettich Zentrifugen, Föhrenstraße, Tuttlingen, Germany). The supernatant was discarded, and the pellet was resuspended in 5 mL of DPBS (Sigma-Aldrich, St. Louis, MO, USA). A viable cell count was carried out on unattached cells by mixing 20 µL of cell suspension with 20 µL of Trypan blue (Sigma-Aldrich, St. Louis, MO, USA) and counting in a hemocytometer (Optik Labor, 0.100 mm, Görlitz, Germany) using a light microscope (Olympus CK40, Tokyo, Japan). Only those cells that were in the four zones of the hemocytometer were counted. Both experiments were performed in triplicate. The percentage of cell attachment was determined using the following equation:

$$\mathrm{Cell}\;\mathrm{attachment}\;(\%)=\frac{\mathrm{Ni}-\mathrm{Nd}}{\mathrm{Ni}}\times 100$$

where Ni is the number of initial cells seeding and Nd is the number of cells in DPBS.

### 2.14. Statistical Analysis

Statistical analysis was performed using GraphPad Prism (Version 5.0; GraphPad Software Inc., San Diego, CA, USA). For multiple groups’ comparison, a two-way variance analysis was used. Statistical significance was set at *p* < 0.05.

## 3. Results

### 3.1. Gross Appearances and Microporous Structure

**Figure 1:**
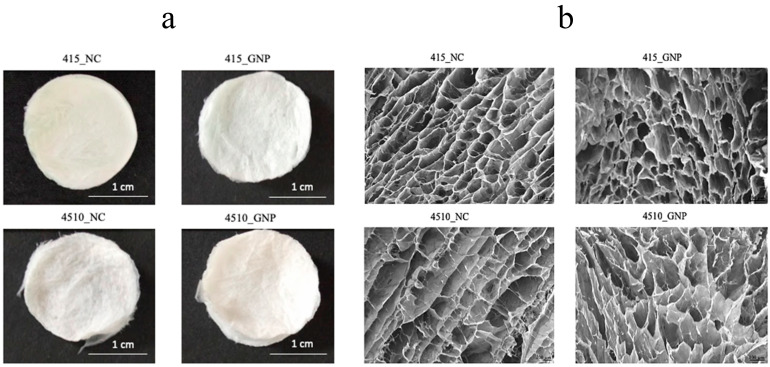

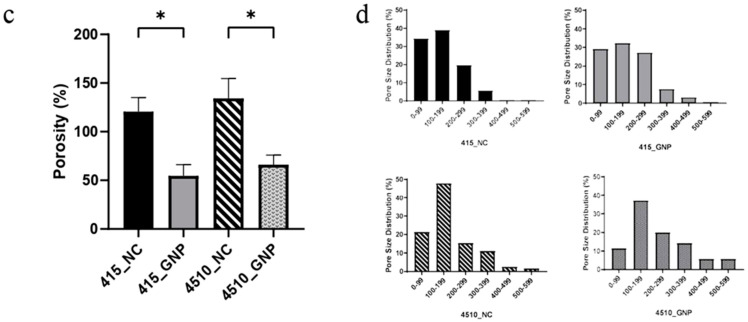
Effect of cross-linking on the collagen–gelatin–elastin (CollaGee) biomaterials. (**a**) Gross appearances of non-cross-linked (NC) 415 (415_NC), non-cross-linked 4510 (4510_NC), genipin (GNP) cross-linked 415 (415_GNP), and GNP cross-linked 4510 (4510_GNP). (**b**) The heterogeneous microstructure of CollaGee sponges. (**c**) Porosity of CollaGee biomaterials revealed that non-cross-linked groups had more interconnected pores than cross-linked groups. (**d**) Pore size distribution of CollaGee biomaterials indicated an acceptable pore size up to 800 µm, with 100–200 µm being dominant in both non-cross-linked and cross-linked groups; * *p* < 0.05.

The gross appearances of both non-cross-linked and cross-linked groups appeared white in color (Figure 1a). FESEM micrographs of the CollaGee biomaterials revealed that the heterogeneous porosity was interconnected (Figure 1b). There was no particle agglomeration detected. Both 415_GNP and 4510_GNP exhibited more than 50% porosity, with no significant difference between the cross-linked groups (Figure 1c). The quantitative measurement of pore sizes (Figure 1d), using Image J software (Version 1.53; National Institutes of Health, Bethesda, MD, USA), demonstrated that 100–199 µm pore size was dominant in all GNP and NC groups.

### 3.2. Mechanical and Physical Behavior

**Figure 2:**
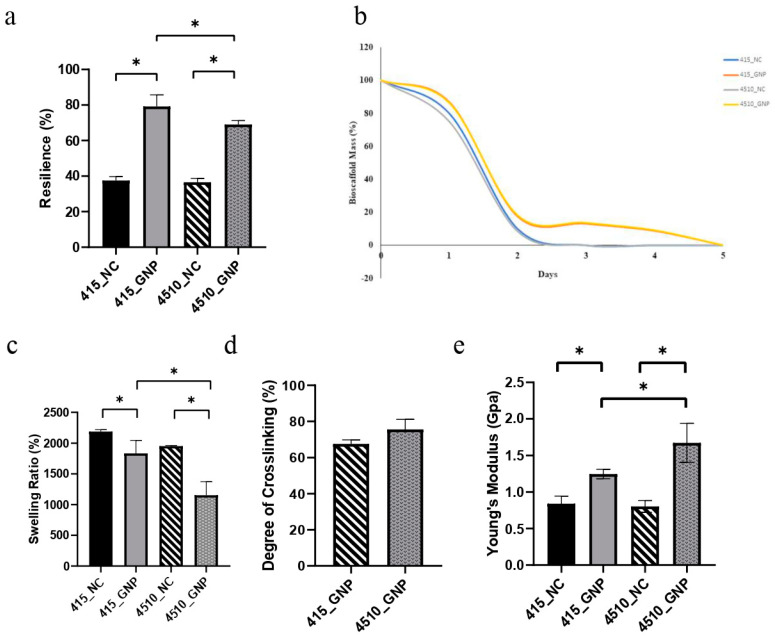
Effect of cross-linking on the physicochemical properties of CollaGee biomaterials. (**a**) Cross-linked groups demonstrated higher resilience than non-cross-linked groups. (**b**) Biodegradation rate showed cross-linked groups had a low biodegradation rate that was good for tissue regeneration. (**c**) Cross-linked groups had a swelling ratio of more than 1000%. (**d**) Post-cross-linking showed more than a 60% cross-linking degree. (**e**) Cross-linked groups had higher mechanical strength than non-cross-linked groups. The 4510_GNP had significantly higher mechanical strength than did 415_GNP. * *p* < 0.05.

Both GNP groups demonstrated significantly higher resilience compared to the NC groups, with a resilience value of more than 60% (Figure 2a). Comparison within the GNP groups found that 415_GNP had significantly higher resilience than 4510_GNP. According to Figure 2b, both cross-linked groups were fully degraded within 5 days and non-cross-linked groups were degraded entirely within 3 days.

All CollaGee biomaterials showed more than 1000% water absorption (Figure 2c), with 415_GNP having a significantly higher swelling capacity than 4510_GNP. In the post-cross-linking with 0.1% GNP, both 415_GNP and 4510_GNP demonstrated more than a 60% cross-linking degree (Figure 2d). There was no significant difference in the cross-linking degrees between both GNP groups. The mechanical strength of the CollaGee biomaterials was evaluated based on Young’s modulus. The GNP groups demonstrated a significantly higher Young’s modulus than the NC groups, with 4510_GNP exhibiting a higher Young’s modulus than the 415_GNP (Figure 2e).

### 3.3. Chemical Characterization

**Figure 3:**
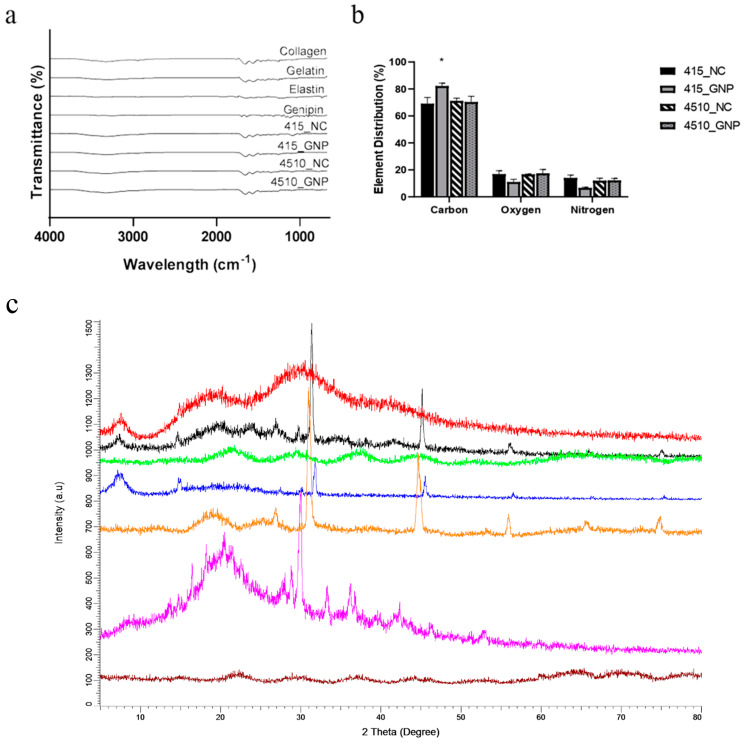
Effect of cross-linking on the chemical and thermal properties of CollaGee biomaterials. (**a**) Fourier-transform infrared spectroscopy (FTIR) analysis to identify chemical structure of CollaGee biomaterials. Results indicated that both 415_GNP and 4510_GNP had no change of chemical structure after cross-linking. (**b**) Energy-dispersive X-ray (EDX) analysis revealed the carbon element was dominant in both cross-linked and non-cross-linked groups, followed by oxygen and nitrogen. Carbon in 415_GNP was significantly higher than in the three remaining groups; * *p* < 0.05. (**c**) X-ray diffraction (XRD) analysis to determine the crystallinity of CollaGee biomaterials. Results indicated that both cross-linked and non-cross-linked groups were amorphous. The spectra from above downwards; 415_GNP, 415_NC, 4510_GNP, 4510_NC, collagen type I, gelatin, and elastin.

The FTIR spectra of non-cross-linked and cross-linked groups showed similar absorbance, resembling the Amide A (3318–3298 cm^−1^), Amide I (1632–1628 cm^−1^), Amide II (1551–1545 cm^−1^), and Amide III (1285–1238 cm^−1^) (Figure 3a). No major shift was prominent in the FTIR spectra after GNP modification. The representative EDX analysis of the electron image of the CollaGee biomaterials exhibited three major elements of carbon, oxygen, and nitrogen (Figure 3b). The XRD results of the CollaGee biomaterials, ovine tendon collagen type I (OTC-1), gelatin, and elastin are demonstrated in Figure 3c. The presence of a prominent peak on 2θ (2Theta) = 7° described the collagen structure sustainability. The XRD spectra of GNP groups showed a broad peak at 2θ = 20–30° angle, indicating the interchain spacing of the collagen triple helix. The results confirmed the amorphous nature of the GNP groups rather than the NC groups.

### 3.4. Thermal Analysis

**Figure 4:**
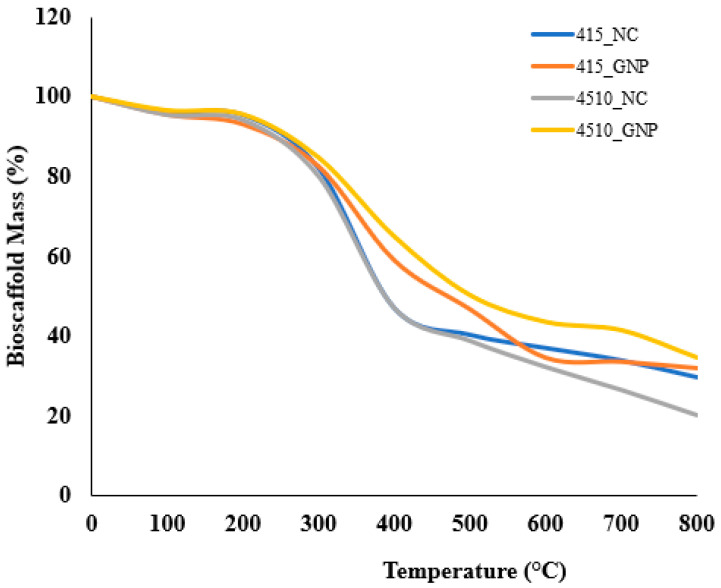
Thermogravimetric analysis (TGA) of CollaGee biomaterials.

The thermal stability of each tested CollaGee biomaterial via DSC is shown in Table 1. The results showed the peak temperatures (Tp) of GNP groups 415_GNP and 4510_GNP (81.70 °C and 99.75 °C) to be higher than NC groups 415_NC and 4510_NC (69.12 °C and 61.29 °C, respectively). Further confirmation using TGA, as demonstrated in Figure 4, showed weight loss at three different regions of the NC groups, with 415_NC at (1) 37–217 °C, (2) 219–664 °C, and (3) 667–797 °C and 4510_NC at (1) 37–202 °C, (2) 204–727 °C, and (3) 729–797 °C. The obtained residues of the CollaGee biomaterials at 780 °C were 29% for 415_NC and 20% for 4510_NC.

After cross-linking with GNP, the biomaterials exhibited a different degradation pattern. In terms of the cross-linked 415_GNP and 4510_GNP, the weight loss process can be divided into four different regions whereby 415_GNP was at (1) 37–199 °C, (2) 202–457 °C, (3) 459–654 °C, and (4) 657–797 °C while 4510_GNP was at (1) 37–185 °C, (2) 187–437 °C, (3) 440–667 °C, and (4) 670–797 °C. The obtained residues of the CollaGee cross-linked biomaterials at 780 °C were 32% for 415_GNP and 34% for 4510_GNP. The NC biomaterials lost approximately 90% of their weight at around 780 °C. In contrast, the addition of GNP into the CollaGee biomaterials presented as more thermally stable with an elevated degradation temperature.

### 3.5. Cell Attachment, Viability, and Biocompatibility

**Figure 5:**
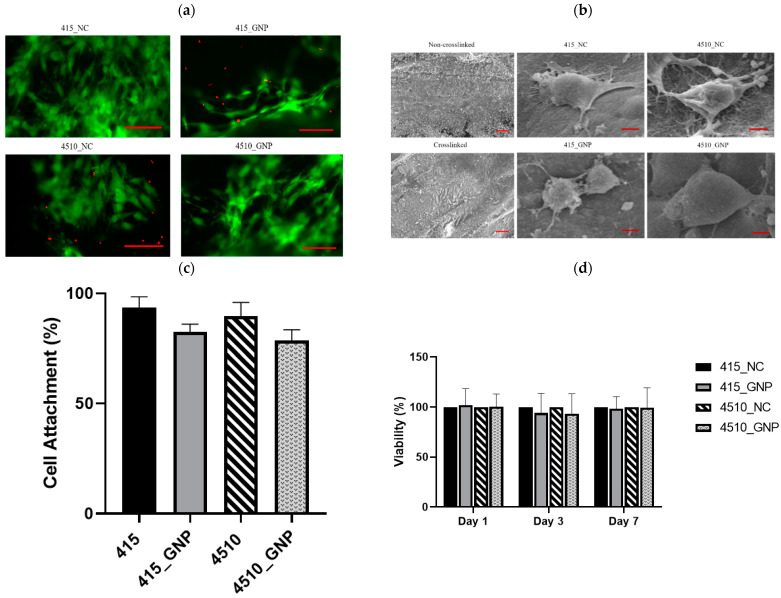
Effect of cross-linking on the cellular biocompatibility of CollaGee biomaterials. (**a**) Live and dead assay, scale bar: 100 µm (**b**) and scanning electron microscope (SEM) analysis, scale bar: 1 µm. The left top and bottom pictures are surface topography without cells, scale bar: 100 µm (**c**) Cell attachment assay revealed more than 80% cell attachments and (**d**) MTT assay revealed no toxicity towards human dermal fibroblasts (HDFs) cultured on cross-linked CollaGee sponges.

The LIVE/DEAD^®^ Cell Viability Assay of HDFs showed green appearances post-seeding on the CollaGee biomaterials, which demonstrated a non-toxic effect (Figure 5a). SEM analysis of HDFs demonstrated that the HDFs seeded onto the CollaGee biomaterials displayed characteristics of round to spindle-shaped morphology with good attachment, extending the HDFs cytoskeleton (Figure 5b). The results of the cell attachment assay of HDFs towards the CollaGee bioscaffolds using Trypan blue exclusion assay via direct method revealed more than 80% cell attachment (Figure 5c). All cross-linked and non-cross-linked groups supported cell proliferation throughout the 7 days of incubation (Figure 5d).

## 4. Discussion

The limitations of certain currently available collagen products on the market are primarily due to the high risk of infection such as bovine spongiform encephalopathy (mad cow disease) and religious constraints such as with porcine products. Therefore, exploring alternative collagen type I (Col-I) sources such as sheep is currently being established and scrutinized. Col-I from ovine tendon can be extracted via salt precipitation, acid-based digestion, or enzymatic isolation using a neutral saline solution or acidic solution such as acetic acid, hydrochloric acid, and acid–enzyme mixtures such as pepsin with acetic acid [32]. The selection of extraction methods depends on the material solubility to produce a high yield of purified Col-I. A low concentration of acetic acid as a green chemical footprint approach is favorable in extracting Col-I from mammals and non-mammals. Acetic acid cleaves the cross-link in collagen and solubilizes the unbound collagen. Meanwhile, a salt solution of sodium chloride (NaCl) is used to precipitate collagen from an acidic solution prior to dialysis [24,32].

The key determining factors to develop the functional biomaterial are the microporous structure, good mechanical strength, biodegradation rate, and biocompatibility [35]. In addition, the porosity of the biomaterial is an essential aspect to evaluate the performance of skin tissue engineering development. Skin biomaterial must be highly porous to transport nutrients and remove wound exudates, hence facilitating cell proliferation and vascularization. Pore sizes between 100 and 300 µm are recommended for cell migration, extensive vascularization, and tissue regeneration [35,36]. Therefore, developing an acellular skin substitute (ASS) for future skin wound-healing treatment is crucial to cater to the need for ready-to-use products for clinicians and surgeons. The abovementioned factors should comply with any natural-based materials’ development for an ideal ASS or wound dressing. In addition, to prolong the sustainability of a cutaneous biomaterial via a freeze-drying/lyophilization approach and/or cross-linking agents, chemicals and irradiation could reduce the risk of commercial loss in the future. The biomaterials’ hybridization approach may affect the native performance of each material; therefore, physicochemical, mechanical, and cellular compatibility assessments are required to validate its behavior.

Lyophilization technology is a well-established and straightforward method to produce porous microstructures, often in sponge-like 3D structures. This process involves the sublimation step to allow the slow removal of the solvent used inside the polymer mixture via lyophilization under high vacuum conditions. This versatile technology enables controlling the mechanical strength, pore size and distribution, porosity, and pore interconnectivity by modifying the polymer concentration and freeze-drying parameters [24]. A previous study comparing different drying methods including oven drying, room temperature drying, and freeze-drying revealed the importance of freeze-drying in forming a porous biomaterial. In this study, freeze-dried CollaGee biomaterials achieved optimum pore sizes around 100–200 µm (Figure 1b) and >50% (Figure 1c) porosity, which is capable of supporting cell migration to accelerate wound healing. However, oven and room temperature drying could not form porous structures, despite their superior drying rate and tensile strength [37]. Thus, the freeze-drying approach is still eligible for fabricating a functionalized porous biomaterial for tissue-engineering applications [38].

The findings revealed that the fabrication of a 3D sponge biomaterial using collagen–gelatin–elastin cross-linked with GNP provides good architecture and physical and mechanical properties and demonstrated no toxicity toward HDFs. As no significant agglomeration of particles was observed on FESEM (Figure 1b), pre-mixing fabrication using a magnetic stirrer (300–450 rpm) presented a good dispersion of biomaterials, with no sedimentation processes occurring during the preparation stage prior to the freeze-drying step. It was further confirmed by EDX analysis, whereby element distribution was homogeneous on different planes (Figure 3b). This confirmed a proper fabrication technique. Furthermore, the XRD’s peaks’ analysis, to determine the crystallography of the fabricated biomaterial and CollaGee, confirmed their amorphous phase resembling native collagen, gelatin, and elastin properties (Figure 3c). Thus, the current pre-mixing fabrication method was proven successful in conserving its original characteristics.

The CollaGee biomaterials generally have poor mechanical strength (Figure 2e); hence, it is necessary to confer structural stability of the biomaterial by introducing exogenous cross-linking agents. This study used natural GNP to cross-link the CollaGee biomaterials due to a more negligible toxic effect on human skin cells. It showed that cross-linked CollaGee presented a higher mechanical strength compared to NC CollaGee (Figure 2e). Fauzi et al. demonstrated a high mechanical strength through GNP cross-linking without affecting the degree of porosity [35]. GNP can form intramolecular and intermolecular cross-linking networks with collagen involving amino acid residues such as lysine, hydroxylysine, and arginine [33]. The cross-linking mechanism for the GNP–amino group monomer was formed through a nucleophilic attack by the amino group-containing compounds on the third carbon of GNP. Subsequently, the resulting aldehyde group was attacked by the attached secondary amino group. Dimerization occurred at the second stage, probably through radical reaction. Therefore, GNP may form intramolecular and intermolecular cross-linked products with a heterocyclic structure [24,32].

Resilience is the ability of a biomaterial to rehydrate into its original form after being loaded with weight. The findings proved that the elasticity and macroporous nature of the biomaterial (Figure 2a) allowed water to freely flow in and out, thereby facilitating cell migration to the wound sites [39]. Additionally, the swelling capacity of a biomaterial is an important aspect to evaluate the performance of cutaneous tissue engineering. A higher swelling capacity will facilitate wound healing as better diffusion of signaling molecules and nutrients will accelerate cell proliferation [40]. The 415_GNP had a significantly higher swelling capacity compared to 4510_GNP (Figure 2c), which could have been due to a higher percentage of gelatin in 4510_GNP that acted as a water-retaining agent. These data are consistent with a previous study performed by Chloe et al. (2012), in which a collagen–gelatin scaffold had more fluid uptake than biomaterial without gelatin [41]. These results demonstrated that GNP is a favorable cross-linking agent for the CollaGee biomaterials since it can efficiently cross-link the amino groups even at a low concentration (0.1% *w*/*v*).

In vitro biodegradation rates were experimented with, using 0.0006% collagenase type I because it resembles the skin microenvironment. Cross-linking with GNP led to the reduction in free amino groups in the hydrolyzed collagen; hence, it may be accountable for a slower degradation rate than the non-cross-linked biomaterials (Figure 2b) [35,42]. This trend may be helpful for skin regeneration in the wound-healing process because faster degradation will lead to a loss of scaffold before a new matrix structure is developed.

Thermal stability, an important feature of biomaterials, means the long-term usability and stability of a biomaterial in extreme environments. The long-term application of regenerative biomaterials at body temperature can lead to damage if the material is thermally unstable [43]. The endothermic peak is the result of a change of structure to a random coil due to the thermal disruption of hydrogen bonds [44]. Consistent with Miles et al., cross-linked groups are more thermally stable than the non-cross-linked groups (Table 1) [45]. The non-cross-linked groups have larger enthalpy than the cross-linked groups due to the presence of non-cross-linked collagen triple helices. An increase in the elastin did not affect the endothermic peak of the biomaterial [46]. Cross-linked CollaGee biomaterials showed water loss at a lower heating temperature, which is consistent with a study performed by Shah et al. [47]. In the first stage, 6 ± 1% of weight loss was observed as structurally bonded water was removed, while a maximum weight loss was seen in the second stage, probably due to chain cleavage of the organic components [48]. The structural characteristic of the collagen family is the right-handed triple helix of three polypeptide chains [24]. A weight loss before 400 °C is commonly due to complex processes such as the dehydration of the polysaccharide rings with the vaporization and removal of volatile products [46]. In the final stage, weight loss was noted to decrease slowly, and a weight loss of only up to 7% was measured (Figure 4). It could be concluded that the water content and thermal stability of the polymers were greatly influenced by the cross-linking degree and intermolecular chain interaction.

An ideal biomaterial for wound healing should support cell viability and migration inside the biomaterial to proliferate and regenerate new tissue [41,46]. In this study, cross-linking with GNP improved the mechanical strength of the biomaterial with negligible cytotoxic effects toward HDFs (Figure 5a–d), which is consistent with previous studies [35,49]. A healthy condition of the HDFs via SEM was characterized by various-shape morphologies with a temporary projection of pseudopodia and excreted matrices (whitish granules) present in the surrounding attached HDFs. Cell attachment of more than 80% and viability of more than 60% proved the non-cytotoxicity of the CollaGee bioscaffolds and a good attachment surface of the HDFs (Figure 5c,d). In addition, an important similarity between collagen and gelatin is the lack or deficient presence of the aromatic amino acids, tryptophan, tyrosine, and phenylalanine. The lack of these amino acids is one of the major contributing factors to the low antigenicity and toxicity of both gelatin and collagen [27]. In addition, the blending of elastin with collagen and gelatin was proven to promote fibroblast proliferation in vitro [3].

## 5. Conclusions

The exploration for better physicochemical, mechanical, and biocompatible properties of biomaterial resembling the native ECM tissues was extensively explored. The use of GNP was demonstrated to contribute effectively to the microstructural, physical, and thermal properties of the CollaGee biomaterials. Further evaluations are needed prior to a preclinical model for efficient study. The use of ASS consisting of collagen, gelatin, and elastin cross-linked with GNP could be explored for the future rapid management of full-thickness skin wounds.

## Figures and Tables

**Table 1:** Thermal transition temperatures of different groups of CollaGee biomaterials. *To*, onset temperature; *Tp*, the temperature of the scaffold peak.

CollaGee Bioscaffolds	Thermal Transition Temperature (°C)	
	*To*	*Tp*
415_NC	64.35	69.12
415_GNP	76.89	81.70
4510_NC	57.37	61.29
4510_GNP	92.50	99.75

## Data Availability

The data presented in this study are available on request from the corresponding author.

## References

[1] Mari C.E., Raquel H.-M., Leire I., Jose L.P., Rajamani L., Alireza D.-P., Nayere T., Gorka O. (2019). Recent advances in gelatin-based therapeutics. Expert Opin. Biol. Ther..

[2] Nike D.U., Abid N., Haliza K., Ruszymah B.H.I., Mh B.F. (2020). Molecular Action of Hydroxytyrosol in Wound Healing: An In Vitro Evidence-Based Review. Biomolecules.

[3] Qingyun W., Suzanne M.M., Anthony S.W. (2020). Elastin Biomaterials in Dermal Repair. Trends Biotechnol..

[4] Ali S., Abid N., Ng M.H., Kok-Yong C., Izhar A.A., Mh B.F. (2020). Natural 3D-Printed Bioinks for Skin Regeneration and Wound Healing: A Systematic Review. Polymers.

[5] Nusaibah S., Abid N., Ruszymah B.H.I., Mh B.F. (2020). Nigella sativa and Its Active Compound, Thymoquinone, Accelerate Wound Healing in an In Vivo Animal Model: A Comprehensive Review. Int. J. Environ. Res. Pub. Health.

[6] Artem A., Naoki M., Norizaku K., Satoru T., Katsuya K., Yuki S., Tsuguyoshi T., Shigehiko S. (2011). Collagen-gelatin scaffold impregnated with bFGF accelerates palatal wound healing of palatal mucosa in dogs. J. Surg. Res..

[7] Chizuru J., Naoki M., Ran I., Michiharu S., Shuichi O., Tsuguyoshi T., Shigehiko S. (2016). A comparison of conventional collagen sponge and collagen-gelatin sponge in wound healing. Biomed. Res. Int..

[8] Jessica F.A., Steven G.W., Anthony S.W. (2012). Elastin signaling in wound repair. Birth Defects Res. C Embryo Today.

[9] Benjamin R.F., David J.M. (2019). Biomaterials to Mimic and Heal Connective Tissues. Adv. Mater..

[10] Nour S., Baheiraei N., Imani R., Khodaei M., Alizadeh A., Rabiee N., Moazzeni S.M. (2019). A review of accelerated wound healing approaches: Biomaterial-assisted tissue remodeling. J. Mater. Sci. Mater. Med..

[11] Alexandra G.-P., Ana-Maria S., Oana C. (2019). Natural composite dressings based on collagen, gelatin and plant bioactive compounds for wound healing: A review. Int. J. Biol. Macromol..

[12] Sheehy E.J., Cunniffe G.M., O’Brien F.J., Barbosa M., Martins M.C. (2018). Collagen-based biomaterials for tissue regeneration and repair. Peptides and Proteins as Biomaterials for Tissue Regeneration and Repair.

[13] Rodríguez-Cabello J.C., Gonzalez D.T.I., Ibáñez-Fonseca A., Alonso M. (2018). Bioactive scaffolds based on elastin-like materials for wound healing. Adv. Drug Deliv. Rev..

[14] Daamen W.F., Veerkamp J.H., van Hest J.C.M., van Kuppevelt T.H. (2007). Elastin as a biomaterial for tissue engineering. Biomaterials.

[15] David M.-N., Elliot L.C. (2016). Collagen and Elastin Biomaterials for the Fabrication of Engineered Living Tissues. ACS Biomater. Sci. Eng..

[16] Mehdi N., Nurkhuzaiah K., Mohd S.M.Y., Abdul S.B., Salma M.Y. (2019). Isolation, Purification and Characterization of Antioxidative Bioactive Elastin Peptides from Poultry Skin. Food Sci. Anim. Resour..

[17] Senior R.M., Griffin G.L., Mecham R.P., Wrenn D.S., Prasad K.U., Urry D.W. (1984). Val-Gly-Val-Ala-Pro-Gly, a repeating peptide in elastin, is chemotactic for fibroblasts and monocytes. J. Cell Biol..

[18] Senior R.M., Griffin G.L., Mecham R.P. (1982). Chemotactic responses of fibroblasts to tropoelastin and elastin-derived peptides. J. Clin. Investig..

[19] Tajima S., Wachi H., Uemura Y., Okamoto K. (1997). Modulation by elastin peptide VGVAPG of cell proliferation and elastin expression in human skin fibroblasts. Arch. Dermatol. Res..

[20] Brassart B., Fuchs P., Huet E., Alix A.J., Wallach J., Tamburro A.M., Delacoux F., Haye B., Emonard H., Hornebeck W. (2001). Conformational dependence of collagenase (matrix metalloproteinase-1) up-regulation by elastin peptides in cultured fibroblasts. J. Biol. Chem..

[21] Fujimoto N., Tajima S., Ishibashi A. (2000). Elastin peptides induce migration and terminal differentiation of cultured keratinocytes via 67 kDa elastin receptor in vitro: 67 kDa elastin receptor is expressed in the keratinocytes eliminating elastic materials in elastosis perforans serpiginosa. J. Investig. Dermatol..

[22] Satsuki M., Bertrand B., Aleksander H. (2002). Signaling pathways transduced through the elastin receptor facilitate proliferation of arterial smooth muscle cells. J. Biol. Chem..

[23] Arnaud R., Abdel F., Jean-Hubert C., Eric H., Loic V., Sandrine L., Franck A., Claudine S., Michel C., William H. (2005). Elastin-derived peptides enhance angiogenesis by promoting endothelial cell migration and tubulogenesis through upregulation of MT1-MMP. J. Cell Sci..

[24] Chun H.J., Park K., Kim C.-H., Khang G. (2018). Novel Biomaterials for Regenerative Medicine.

[25] Samson A., Amir S., Soundarapandian K., Samad A., Ali K. (2019). Gelatin-polysaccharide composite scaffolds for 3D cell culture and tissue engineering: Towards natural therapeutics. Bioeng. Transl. Med..

[26] Karin V.G., Lydia F., Honglei H., Rutger P., Aldo R.B., Tahera A. (2017). Is quercetin an alternative natural crosslinking agent to genipin for long-term dermal scaffolds implantation?. J. Tissue Eng. Regen. Med..

[27] Alvin B.B., Doegil K., Dohyun K., Hansoo P., Soo-Hong L. (2020). Engineering and Functionalization of Gelatin Biomaterials: From Cell Culture to Medical Applications. Tissue Eng. Part B Rev..

[28] Yi Z., Qiang-Song W., Kuo Y., Yun Q., Gui-Fang W., Yuan-Lu C. (2016). Preparation, characterization, and evaluation of genipin crosslinked chitosan/gelatin three-dimensional scaffolds for liver tissue engineering applications. J. Biomed. Mater. Res. A.

[29] Qin M., Chong S. (2018). Construction of low contracted 3D skin equivalents by genipin cross-linking. Exp. Dermatol..

[30] Huang G.-Q., Han X.-N., Xiao J.-X., Cheng L.-Y. (2016). Effects of coacervation acidity on the genipin crosslinking action and intestine-targeted delivery potency of the O-carboxymethyl chitosan–gum arabic coacervates. Int. J. Polym. Mater..

[31] Mu C., Zhang K., Lin W., Li D. (2013). Ring-opening polymerization of genipin and its long-range crosslinking effect on collagen hydrogel. J. Biomed. Mater. Res..

[32] Fauzi M.B., Yogeswaran L., Aminuddin B.S., Ruszymah B.H.I., Shiplu R.C. (2016). Ovine tendon collagen: Extraction, characterisation and fabrication of thin films for tissue engineering applications. Mater. Sci. Eng. C.

[33] Fauzi M.B., Shiplu R.C., Fuad B.I., Aminuddin B.S., Ruszymah B.H.I. (2016). Fauzi, Tissue-Engineered Skin Substitute Enhances Wound Healing after Radiation Therapy. Adv. Skin Wound Care.

[34] Zarei M., Samimi A., Khorram M., Abdi M.M., Golestaneh S.I. (2021). Fabrication and characterization of conductive polypyrrole/chitosan/collagen electrospun nanofiber scaffold for tissue engineering application. Int. J. Biol. Macromol..

[35] Fauzi M.B., Nor F.R., Yasuhiko T., Aminuddin B.S., Ruszymah B.H.I., Shiplu R.C. (2019). Rapid Treatment of Full-Thickness Skin Loss Using Ovine Tendon Collagen Type I Scaffold with Skin Cells. J. Tissue Eng. Regen. Med..

[36] Loh Q.L., Choong C. (2013). Three-dimensional scaffolds for tissue engineering applications: Role of porosity and pore size. Tissue Eng. Part B Rev..

[37] Liu B.S., Yao C.H., Wang W., Lee S., Lo C.C., Liu C.K., Chen Y.S. (2008). Effect of drying method on the characteristics of genipin cross-linked gelatin films. J. Med. Biol Eng..

[38] Muhammad M.A.A., Mohd H.M.Y., Mh B.F. (2019). Genipin-Crosslinked Gelatin Scaffold in Tissue Engineering: A Systematic Review. Med. Health.

[39] Liu Y., Xu K., Chang Q., Mohammad A.D., Bojie L., Wen Z., Malcolm X. (2016). Highly Flexible and Resilient Elastin Hybrid Cryogels with Shape Memory, Injectability, Conductivity, and Magnetic Responsive Properties. Adv. Mater..

[40] Sánchez P., Pedraz J.L., Orive G. (2017). Biologically active and biomimetic dual gelatin scaffolds for tissue engineering. Int. J. Biol. Macromol..

[41] Chloe N.G., Ruth E.C., Serena M.B. (2012). Investigating the morphological, mechanical and degradation properties of scaffolds comprising collagen, gelatin and elastin for use in soft tissue engineering. J. Mech. Behav. Biomed. Mater..

[42] Liang H.C., Chang W.H., Liang H.F., Lee M.H., Sung H.W. (2004). Crosslinking structures of gelatin hydrogels crosslinked with genipin or a water-soluble carbodiimide. J. Appl. Polym. Sci..

[43] Yavuz E.A., Tugba S.A., Burak D., Emel E., Kaan C.E. (2017). Fabrication of human hair keratin/jellyfish collagen/eggshell-derived hydroxyapatite osteoinductive biocomposite scaffolds for bone tissue engineering: From waste to regenerative medicine products. Colloids Surf. B Biointerfaces.

[44] Skopinska-Wisniewska J., Kuderko J., Bajek A., Maj M., Sionkowska A., Ziegler-Borowska M. (2016). Collagen/elastin hydrogels cross-linked by squaric acid. Mater. Sci. Eng. C.

[45] Christopher A.M., Nicholas C.A., Victor V.R., Allen J.B. (2005). The increase in denaturation temperature following cross-linking of collagen is caused by dehydration of the fibers. J. Mol. Biol..

[46] Koji T., Makoto H. (1993). Edible Meat Casing from Reconstruction of Collagen-Elastin Matrix. J. Food Sci..

[47] Rushita S., Pavel S., Katerina S., Petr S. (2019). Dual Crosslinked Collagen/Chitosan Film for Potential Biomedical Applications. Polymers.

[48] Nawshad M., Girma G., Abdur R., Pervaiz A., Farasat I., Faiza S., Amir S.K., Farman U.K., Zia U.H.K., Fozia R. (2017). Investigation of ionic liquids as a pretreatment solvent for extraction of collagen biopolymer from waste fish scales using COSMO-RS and experiment. J. Mol. Liq..

[49] Mina M., Kenneth K.H.W., Donna T.P., Yan W., David B.O., Wankei W. (2011). Genipin-cross-linked electrospun collagen fibers. J. Biomater. Sci..

